# A low concentration of choline chloride alters the developmental program of the bovine preimplantation embryo

**DOI:** 10.1530/RAF-24-0058

**Published:** 2024-10-25

**Authors:** McKenzie L J Haimon, Eliab Estrada-Cortés, Thiago Fernandes Amaral, Jeremy Block, Surawich Jeensuk, Tatiane S Maia, Quinn A Hoorn,, Masroor Sagheer,, João H Bittar, Peter J Hansen

**Affiliations:** 1 Department of Animal Sciences, University of Florida, Gainesville, Florida, USA; 2 Campo Experimental Centro Altos de Jalisco, Instituto Nacional de Investigaciones Forestales, Agrícolas y Pecuarias, Tepatitlán de Morelos, Jalisco, México; 3 Department of Animal Science, University of Wyoming, Laramie, Wyoming, USA; 4 Department of Livestock Development, Bureau of Biotechnology in Livestock Production, Pathum Thani, Thailand; 5 Department of Large Animal Medicine, College of Veterinary Medicine, University of Florida, Gainesville, Florida, USA

**Keywords:** Cattle, choline, developmental programming, methyl donor, preimplantation embryo, weaning

## Abstract

Choline is a known developmental programming agent of the bovine preimplantation embryo. Culture of the embryo with 1.8 mmol/L choline, a concentration much higher than in blood, alters development to cause increased weaning weight and other changes during the postnatal period. It was hypothesized here that choline exerts similar effects on the developmental program of the embryo when added at concentrations similar to those in peripheral blood (i.e. 4 µmol/L). Oocytes were collected via ovum pickup, and embryos were produced *in vitro*. Embryos were cultured until day 7 after fertilization in medium with 4 µmol/L choline chloride or, as a vehicle control, with an additional 4 µmol/L sodium chloride. Blastocysts were transferred into recipients, and pregnancy was diagnosed at approximately 28 days of gestation. Subsequent calves (*n* = 37 for vehicle and *n* = 35 for choline) were weighed at birth and at weaning. Addition of choline to the culture medium did not affect the proportion of embryos that became blastocysts or the proportion of transferred blastocysts that produced a pregnancy. Birth weight was unaffected by treatment, but calves derived from choline-treated embryos were heavier at the time of weaning and gained more per day from birth until weaning than calves derived from embryos treated with vehicle. Results demonstrate that choline can act on the preimplantation embryo at a physiologically relevant concentration to alter postnatal phenotype. Observations are further evidence for the importance of the first days of embryonic development for the phenotype of the resulting calf.

**Lay summary:**

The period of early embryonic development is one in which changes in the environment of the embryo can alter its development and affect characteristics of the resultant offspring. Here, we show that providing a nutrient called choline to the cow embryo for the first 7 days of development increased growth after calving. These results suggest that it may be possible to program embryonic development early in pregnancy to improve livestock production.

## Introduction

One-carbon metabolism refers to a series of metabolic pathways that provide methyl groups for biosynthesis, methylation of DNA and histones for epigenetic regulation, and redox balance. Among the key enzymatic systems involved in one-carbon metabolism are those driving the folate and methionine cycles. The importance of one-carbon metabolism for the preimplantation embryo is indicated by studies showing that the addition of the folate antagonist methotrexate blocked embryonic development in cattle and sheep (Kwong *et al.* 2010), while knockdown of mRNA for folate receptor-1 reduced the development of preimplantation goat embryos (Saini *et al.* 2022). Similarly, ethionine, which inhibits methionine metabolism, blocked the development of cultured mice (Menezo *et al.* 1989) and bovine embryos (Ikeda *et al.* 2012), as did an inhibitor of methionine adenosyltransferase, which converts methionine to S-adenosylmethionine (Ikeda *et al.* 2017).

It is likely that alterations in the availability of key nutrients involved in one-carbon metabolism can cause epigenetic alterations in the preimplantation embryo and long-term changes in the developmental program of the resultant fetus. The embryo undergoes extensive epigenetic remodeling during the preimplantation period, including the removal and subsequent gene- and cell-type specific acquisition of DNA methylcytosines, as well as changes in the abundance and location of specific methylation marks on histones (Xu *et al.* 2021, Zhu *et al.* 2021). Sinclair *et al.* (2007) fed ewes a diet deficient in cobalt and sulfur from 8 weeks before to 6 days after conception to reduce rumen microorganism capacity for the synthesis of sulfur-containing amino acids and vitamin B_12_. The resultant lambs were heavier and fatter than controls and had elevated blood pressure and altered immune and insulin responsiveness. *In vitro,* alterations in concentrations of molecules involved in one-carbon metabolism, such as methionine (Clare *et al.* 2021), S-adenosylmethionine (Shojaei Saadi *et al.* 2016), or homocysteine (Ikeda *et al.* 2010), altered DNA methylation in the preimplantation embryo.

Recent research using the bovine as a model suggests that the quaternary amine choline can also program the development of the preimplantation embryo in a way that alters postnatal phenotype. Choline can function as a methyl donor through its enzymatic conversion to betaine, methionine, and S-adenosylmethionine. It also serves as a precursor to phosphatidylcholine and acetylcholine and can induce cell signaling indirectly through S-adenosylmethionine-induced activation of mTOR signaling (Gu *et al.* 2017). The culture of embryos with 1.8 mmol/L choline chloride increased DNA methylation in the resultant blastocysts (Estrada-Cortés *et al.* 2020). Calves derived from blastocysts cultured with 1.8 mmol/L choline experienced a variety of postnatal changes compared with calves derived from embryos cultured without choline, including increased weaning weight and carcass weight, as well as changes in DNA methylation in skeletal muscle and white blood cells (Estrada-Cortés *et al.* 2021*a*, Haimon *et al.* 2024).

The aim of the present study was to test whether a concentration of choline chloride similar to those found in circulation during the postpartum period was sufficient to program the preimplantation embryo to affect the postnatal phenotype. The concentration of choline chloride that altered the developmental program of the embryo to affect postnatal phenotype, 1.8 mmol/L (Estrada-Cortés *et al.* 2021*a*, Haimon *et al.* 2024), was based on the sum of concentrations of all choline metabolites in the blood of postpartum dairy cows fed rumen-protected choline (Estrada-Cortés *et al.* 2020). The actual concentration of choline chloride in the blood of postpartum dairy cows is much lower, with average values in blood plasma in cows during the first 13 weeks of lactation varying from 3.75 to 4.47 µmol/L (Artegoitia *et al.* 2014). Accordingly, bovine zygotes were cultured in a medium with and without 4 µmol/L choline chloride, blastocysts were transferred into recipient females, and the characteristics of the resultant calves were determined through weaning.

## Materials and methods

### Overview

**Figure 1:**
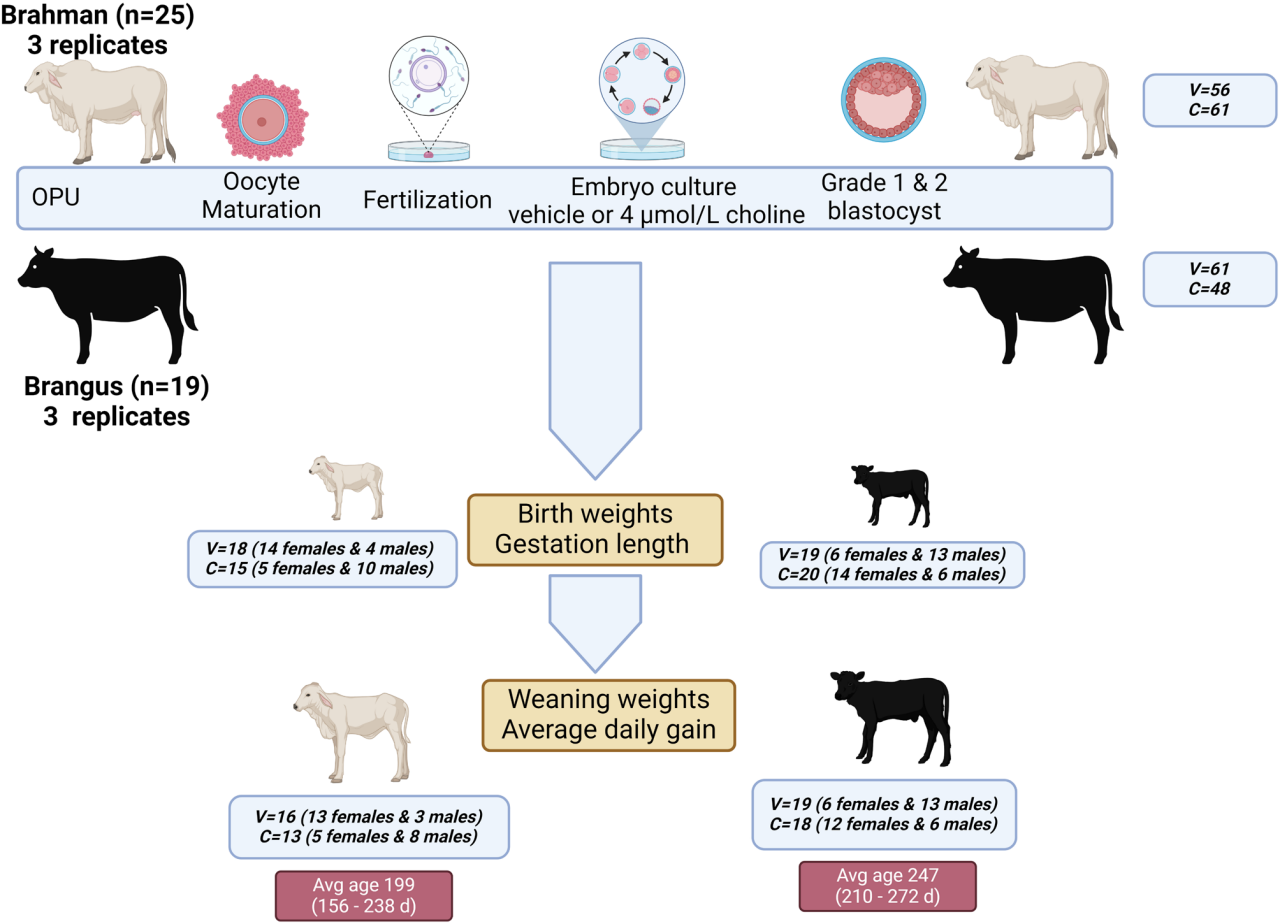
Experimental design. Two farms were utilized in the experiment, with one farm using Brahman cattle (*n* = 25 donors) and the other using Brangus cattle (*n* = 19 donors). Each donor underwent ovum pick-up (OPU) a total of one to three times. Oocytes were used for *in vitro* fertilization. For each round of OPU, all presumptive zygotes from each donor were randomly assigned to culture with 4 µmol/L choline or the vehicle for 7 days. Grade 1 and 2 blastocysts were transferred to recipients. Calves were weighed at birth and at weaning at an average age of 199 days (Brahman) or 247 days (Brangus). V = number of vehicle animals and C = number of choline animals.

The experiment was approved by the Animal Care and Use Committee at the University of Florida. An overview of the experimental design is provided in Fig. 1. The experiment was conducted at two locations (Gainesville, Florida – Brahman; Fort Pierce, Florida – Brangus) in six replicates of transfering *in vitro-*produced embryos cultured with 0 or 4 µmol/L choline chloride into recipient females. Three replicates involved the transfer of 117 Brahman embryos into crossbred recipients (composed of various admixtures of Brahman and Angus genetics), while three replicates involved the transfer of 109 Brangus embryos into Brangus recipients. Transferrable embryos were produced using cumulus-oocyte complexes (COCs) collected via ultrasound-guided transvaginal ovum pick-up (OPU) from 25 Brahman cows (*n* = 21 cows with one round of OPU, *n* = 3 with two rounds, and *n* = 1 with three rounds) and 19 Brangus cows (*n* = 16 cows with one round of OPU and *n* = 3 cows with two rounds of OPU). The total number of sires used was 15 (12 Brahman and 3 Brangus). All semen was conventional, except for one Brahman sire, whose semen was X-sorted. For each OPU, oocyte donors were randomly assigned to one of two treatments, 4 µmol/L additional sodium chloride (called vehicle) or 4 µmol/L choline chloride. All presumptive zygotes from one donor received one treatment. For donors used for more than one OPU session, treatment was assigned randomly for each replicate. At ~day 7, blastocysts were transferred to recipients who were randomly assigned to treatment. Recipients were diagnosed as pregnant at 28 days of gestation and housed together for the duration of gestation. The subsequent calves were weighed at birth and at weaning. The number of calves derived from embryo transfer included 33 Brahman calves (18 from embryos treated with vehicle and 15 from embryos treated with choline chloride) and 39 Brangus calves (19 from embryos treated with vehicle and 20 from embryos treated with choline). Calves were produced from nine sires and 15 dams (Brahman), and three sires and 14 dams (Brangus). Researchers knew which embryos were treated with choline or the vehicle, but other assignments were blinded with respect to personnel performing embryo transfers, providing care to experimental animals, or collecting data.

### Production of embryos

All embryos were produced *in vitro* utilizing oocytes collected via OPU as described elsewhere (Estrada-Cortés *et al.* 2021*b*). Donor animals did not receive any hormonal treatments prior to oocyte aspiration. Visible follicles were punctured and aspirated for each donor. Aspiration fluid was filtered and all COCs were sorted and placed in groups of up to 30 in 1.5 mL maturation medium (Boviteq, Madison, WI, USA). Details of oocyte maturation, fertilization, and embryo culture are described elsewhere (Tríbulo *et al.* 2019*b*, Estrada-Cortés *et al.* 2021*b*). Briefly, oocytes were matured for 22–23 h at 38.5°C. The COCs were then washed with HEPES-buffered Tyrode’s albumin-lactate-pyruvate (HEPES-TALP) and co-cultured with 1 × 10^6^ sperm/mL from a single bull per donor for 10 h at 38.5°C in a humidified atmosphere of 5% (v/v) CO_2_. To prepare sperm for insemination, frozen semen was thawed and purified using PureSperm^TM^ 40/80 gradient (Nidacon International AB, Mölndal, Sweden).

After insemination, presumptive zygotes were placed in a 1.5 mL conical tube containing 1000 IU/mL hyaluronidase and 300 µL of HEPES-TALP and vortexed for 3 min to remove cumulus cells. Presumptive zygotes were then washed with HEPES-TALP and placed in groups of 30 per 45 µL drop of BBH7 (Cooley Biotech, Gainesville, Florida, USA). All presumptive zygotes from an individual donor received one of the two treatments. Donors were assigned randomly to either vehicle or choline treatments. Treatments were prepared in DPBS and added in 5–45 µL BBH7 culture medium to create a final concentration of 4 µmol/L additional sodium chloride (vehicle) or 4 µmol/L choline chloride (choline) and a final volume of 50 µL. Treatments were added at the beginning of embryo culture. Embryos were cultured for 7 days without medium changes or additions.

Cleavage rate was assessed on day 3 after fertilization by evaluating the number of embryos with more than two cells. The number of blastocysts was assessed on day 7 after fertilization. Blastocysts were graded, and those classified as grade 1 (77.6%) or grade 2 (22.4%) (Bó & Mapletoft 2013) were selected for transfer. Individual embryos were loaded into 0.25 mL embryo transfer straws in a holding medium comprised of HEPES-TALP, 10% (v/v) fetal bovine serum, and 50 µmol/L dithiothreitol. Straws were then placed in a portable incubator (Micro Q Technologies, Scottsdale, Arizona, USA) at 38.5°C and transported to the farm for transfer approximately 5–8 h after harvest.

### Embryo transfer

Recipients were cows at least 70 days postpartum. There were 71 lactating and 46 non-lactating Brahman recipients. All 109 Brangus recipients were non-lactating. Ovulation of recipients was synchronized as follows: on day 0, 100 µg of gonadotropin-releasing hormone (GnRH; Cystorelin®, Boehringer Ingelheim, Duluth, GA, USA) was injected i.m., and an intravaginal progesterone-releasing device (CIDR®, Zoetis, Parsippany-Troy Hills, NJ, USA) was inserted. On day 7, the CIDR was removed, and 25 mg of prostaglandin F2a (Lutalyse®, Zoetis) was injected i.m. On day 9, 100 µg GnRH was injected i.m. Embryo transfer was performed on day 17 (i.e. 7.5 days after the presumed day of ovulation) after confirming the presence of a functional corpus luteum via ultrasonography. Recipients received caudal epidural injections of 5 mL 2% (w/v) lidocaine hydrochloride (Aspen Veterinary Resources, Liberty, MO, USA). One blastocyst was transferred into the uterine horn ipsilateral to the corpus luteum. The pregnancy rate was determined at 28 days of gestation.

### Collection of calf data

Recipients from each replicate were housed together through the weaning of calves at an average age of 199 days (range: 156–238 days) for Brahman and 247 days (range: 210–272 days) for Brangus. Calves were weighed using a digital scale at birth and at weaning. Bull calves were kept intact for the entire observational period. The 205-day adjusted weaning weight was calculated as ((weaning weight − birth weight)/days of age) * 205 + birth weight.

### Statistical analysis

SAS version 9.4 (SAS Institute, Cary, NC, USA) was utilized for data analysis. All data points were included in the analysis. Binomial variables, including the proportion of presumptive zygotes cleaving and reaching the blastocyst stage, the proportion of cleaved embryos reaching the blastocyst stage, and pregnancy outcomes, were analyzed by logistic regression fit to a binomial distribution using the GLIMMIX procedure. Effects in the model for development data included treatment, farm, interaction between treatment and farm, replicate nested within the farm, and treatment × replicate interaction, with donor as a random term. Effects in the model for pregnancy outcomes (recipient as the experimental unit) included treatment, farm, the interaction of treatment and farm, sire nested within farm, replicate nested within the farm, and the interaction of treatment and replicate nested within farm. Continuous variables (calf as the experimental unit) were evaluated for normality and analyzed by analysis of variance using the GLM procedure of SAS. Model effects included treatment, farm, replicate nested within the farm, sire nested within the farm, sex of calf, the treatment by farm interaction, the interaction of treatment by replicate nested within the farm, and the treatment by sex interaction. Calf age was used as a covariate for data collected at weaning. All effects of treatment, treatment × sex, and farm × treatment with *P* < 0.10 are noted.

## Results

### Embryo development and pregnancy outcomes after transfer

Effects of choline chloride on cleavage and embryonic development are summarized in Table 1. Choline did not affect the proportion of oocytes that cleaved (*P* = 0.9845) or that became blastocysts at day 6.5 (*P* = 0.3117). Choline also did not affect the percentage of cleaved embryos becoming blastocysts (*P* = 0.1916).

**Table 1:** Effect of 4 µmol/L choline chloride added during the first 7 days of embryo culture on characteristics of *in vitro* fertilization and embryonic development. Data are least-squares means ± s.e.m.

Trait	Vehicle	Choline	*P*
Cleaved oocytes (%)	77.4 ± 0.03	77.5 ± 0.03	0.9845
Blastocysts/oocytes (%)	13.4 ± 0.02	10.9 ± 0.02	0.3117
Blastocysts/cleaved embryos (%)	18.4 ± 0.02	14.4 ± 0.02	0.1916

Pregnancy outcomes after transfer are reported in Table 2. Treatment with choline did not affect pregnancy outcomes at day 28 (*P* = 0.8373) or the proportion of recipients that calved (*P* = 0.9617). There was a numerical difference (farm × treatment; *P* = 0.1630) in the effect of choline on pregnancy outcomes at day 28, which was positive for Brangus (39.3% (24 pregnant recipients/61 total) for vehicle vs 52.1% (25/48) for choline), but not Brahman (35.7% (20/56) vs 29.5% (18/61) for choline). The interaction approached significance (*P* = 0.0549) for the calving rate. Choline increased the calving rate for Brangus (29.5% (18/61) for vehicle vs 43.8% (21/48) for choline) but not for Brahman (30.4% (17/56) vs 26.2% (16/61) for choline). There was also no effect of treatment (*P* = 0.9940) or the interaction between farm and treatment (*P* = 0.9935) on the loss of pregnancies between day 28 and calving. Pregnancy loss for Brangus was 25.0% (6/24) for vehicle vs 16.0% (4/25) for choline. Pregnancy loss for Brahman was 15.0% (3/20) vs 11.1% (2/18) for choline. Only two calves, both Brahman, were stillborn (one choline and one vehicle).

**Table 2:** Effect of 4 µmol/L choline chloride added during the first 7 days of embryo culture on pregnancy outcomes after embryo transfer.

Trait	Vehicle^a^	Choline^a^	*P*
Pregnant at 28 d	44/117 (37.6%)	43/109 (39.4%)	0.8373
Pregnancy loss after 28 d	9/44 (20.5%)	6/43 (13.9%)	0.9940
Calving rate	35/117 (29.9%)	37/109 (33.9%)	0.9617

^a^Data are the fraction and percent of cows.

### Postnatal characteristics of calves from birth to weaning

Data are summarized in Table 3 for both female and male calves. There was no effect of choline treatment on gestation length (*P* = 0.4364) or calf birth weight (*P* = 0.3999). Calves were weaned at an average age of 199 days (range: 156–238 days) for Brahman and 247 days (range: 210–272 days) for Brangus. A total of four calves died between birth and weaning (one vehicle and three choline). Calves derived from choline-treated embryos had increased weaning weight compared to calves derived from vehicle-treated embryos. This was true when examining actual body weight (after including age as a covariate) (*P* = 0.0239) as well as the 205-day adjusted weaning weight used in the US cattle industry to adjust weaning weight for age at weaning (*P* = 0.0050). Average daily gain from birth to weaning was also greater (*P* = 0.0188) for choline than for vehicle. There were no interactions between treatment and the farm. For example, the adjusted weaning weight for Brahman calves was 220.3 ± 7.2 kg for vehicle and 236.0 ± 6.9 kg for choline, while the adjusted weaning weight for Brangus was 194.6 ± 5.2 kg for vehicle and 210.2 ± 5.9 kg for choline.

**Table 3:** Effects of 4 µmol/L choline chloride added during the first 7 days of embryo culture on postnatal characteristics of the calves derived from those embryos. Data are least-squares means ± s.e.m.

Trait	Female		Male		*P*		
	Vehicle^b^	Choline^c^	Vehicle^d^	Choline^e^	Treatment	Sex	Treatment × sex interaction
Gestation length (d)	286.2 ± 1.3	285.2 ± 1.3	286.4 ± 1.5	285.3 ± 1.3	0.4364	0.9083	0.9284
Birth weight (kg)	34.0 ± 1.9	34.4 ± 1.9	35.5 ± 2.2	38.5 ± 1.9	0.3999	0.1592	0.5075
Weight at weaning (kg)^a^	239.0 ± 5.8	242.9 ± 6.1	251.9 ±7.1	278.6 ± 6.0	0.0239	0.0002	0.0645
205-day adjusted weaning weight (kg)^a^	201.8 ± 5.0	210.8 ± 5.0	213.0 ±5.7	235.4 ± 4.8	0.0050	0.0007	0.1766
Average daily gain from birth to weaning (kg/d)^a^	0.91 ± 0.02	0.94 ± 0.02	0.97 ± 0.03	1.1 ± 0.02	0.0188	0.0004	0.1743

^a^Data were collected at weaning at an average age of 199 days (range 156–238 d) for Brahman and 247 days (range 210–272 d). Age at weaning was used as a covariate in the statistical analysis; ^b^*n* = 20 for birth weight and *n* = 19 for other traits; ^c^*n* = 19 for birth weight and *n* = 17 for other traits; ^d^*n* = 17 for birth weight and *n* = 16 for other traits.; ^e^*n* = 16 for birth weight and *n* = 14 for other traits.

There were effects of sex on weaning weight *(P* = 0.0002), 205-day adjusted weaning weight (*P* = 0.0007), and average daily gain (*P* = 0.0004), with males being greater than females. The effect of choline for weaning weight tended to be greater for males than females (treatment × sex; *P* = 0.0645). The interaction was *P* = 0.1766 for adjusted weaning weight.

## Discussion

The results presented here indicate that, as previously reported (Estrada-Cortés *et al.* 2021*a*, Haimon *et al.* 2024), choline chloride can act on the preimplantation bovine embryo to program development in a manner that results in calves with heavier birth weights. Unlike the previous experiments, in which embryos were cultured with 1.8 mmol/L choline chloride, the programming actions of choline chloride in the present study were induced at a concentration of 4 µmol/L, which approximates the concentrations present in the blood of postpartum dairy cows not fed rumen-protected choline (Artegoitia *et al.* 2014). These results lead to the conclusion that choline chloride can program the development of the bovine embryo at a physiologically relevant concentration. It is also noteworthy that the actions of choline chloride were observed using cattle of two separate breeds. The fact that the main alteration in phenotype caused by choline chloride (increased weaning weight) is a trait that is important for the economic outcomes of beef cattle production means that it might be possible to modify choline availability to the bovine embryo to enhance the profitability and sustainability of beef cattle production.

Actions of choline chloride reported here largely reflect those seen in the earlier experiments with higher concentrations of choline (Estrada-Cortés *et al.* 2021*a*, Haimon *et al.* 2024). Like those earlier experiments, there was no effect of choline chloride on the percentage of embryos becoming blastocysts or on pregnancy success after embryo transfer. In one experiment (Estrada-Cortés *et al.* 2021*a*), choline chloride treatment during the preimplantation period resulted in longer gestation lengths and greater birth weights. No effect on these variables was observed here or in another experiment (Haimon *et al.* 2024).

A common phenomenon associated with developmental programming is sexual dimorphism, where the consequences of exposure to a developmental programming agent are different for male offspring than for female offspring. Such a phenomenon often occurs for developmental programming during the preimplantation period (Hansen *et al.* 2016). Here, the actions of choline on weaning weight tended to be more pronounced in male calves than in female calves, but sex differences in response to choline were not significant for adjusted weaning weight or average daily gain. There were no significant interactions between choline and sex on body weight in the earlier experiments (Estrada-Cortés *et al.* 2021*a*, Haimon *et al.* 2024). One of these earlier studies (Estrada-Cortés *et al.* 2021*a*) was underpowered for the examination of a treatment by sex interaction. In the other experiment (Haimon *et al.* 2024), there were no interactions with sex for body weight, but there was a treatment by sex interaction for feed intake and feed efficiency. In particular, choline calves consumed less feed and were more efficient than vehicle calves for females, while the opposite was true for male calves. Furthermore, DNA methylation changes in the white blood cells of calves were sex-dependent. More experimentation is needed to resolve whether the actions of choline chloride to program postnatal phenotype are greater for one sex than the other.

An important question is whether feeding choline chloride during the preimplantation period could cause programming effects similar to those seen here for embryos exposed to choline chloride in culture. In ruminants, dietary choline is degraded by rumen microorganisms, and *de novo* synthesis is the predominant source of choline (Pinotti *et al.* 2002). Formulations of choline have been developed that bypass rumen degradation. Feeding rumen-protected choline can increase milk production (Arshad *et al.* 2020). The results of the current experiment indicate that even a low concentration of choline chloride is sufficient to alter the developmental program of the preimplantation embryo. What is not known is whether feeding rumen-protected choline changes concentrations of choline chloride in the oviduct or uterus by a sufficient amount to alter embryonic function. Indeed, in a recent paper, the provision of an oral bolus of rumen-protected choline to lactating cows caused only slight or no changes in concentrations of choline chloride in blood plasma (France *et al.* 2022). Choline is present in the uterine lumen of cows early after ovulation (Tríbulo *et al.* 2019*a*), but the actual concentration is unknown. Studies to determine optimal feeding methods to increase choline concentrations in reproductive tract fluids are warranted, as are studies evaluating the impact of feeding rumen-protected choline during early pregnancy on the phenotype of the offspring.

Another question is the mechanism by which choline programs develop in the preimplantation period to result in greater postnatal growth. In earlier experiments, choline treatment of embryos caused changes in DNA methylation in muscle (Estrada-Cortés *et al.* 2021*a*) and blood (Haimon *et al.* 2024) of the resultant calves. It is possible that physiological systems involving specific genes that were differentially methylated changed postnatal growth. It is also possible that placental function was modified by choline in a way that altered maternal milk yield after calving. However, there was no effect of choline treatment of the preimplantation embryo on circulating concentrations of pregnancy-associated glycoproteins produced by trophoblast giant cells (Haimon *et al.* 2024).

In conclusion, the addition of choline chloride to cultures of bovine embryos at a concentration similar to that of circulating plasma concentrations is sufficient to program postnatal phenotype, particularly daily weight gain through weaning and weaning weight. These results highlight the potential to modify the developmental program of the embryo through nutritional interventions in the preimplantation period to enhance the sustainability of livestock production.

## Declaration of interest

All authors declare that there were no conflict of interest that could be perceived as prejudicing the impartiality of the study reported.

## Funding

The research was supported by USDA-NIFA grant 2020-67015-30821, USDA Food and Agricultural Sciences National Needs Graduate Fellowships Program 2019-38420-28977 and 2021-38420-34067, the L.E. “Red” Larson Endowment, the Higher Education Council of Pakistan, and the Royal Thai Government.

## Author contribution statement

Experiments were conceived and designed by MLJH and PJH. Experimental procedures were conducted by all authors. Data analysis was performed by MLJH and PJH. The first draft of the paper was written by MLJH, and all authors participated in manuscript editing.
